# Valorization of Cocoa Husks: Pectin Recovery

**DOI:** 10.1155/2019/1212081

**Published:** 2019-04-01

**Authors:** Chiara Mollea, Fulvia Chiampo

**Affiliations:** Department of Applied Science and Technology, Politecnico di Torino, 10129 Torino, Italy

## Abstract

Food processing by-products are usually cheap and abundant and can be source of valuable molecules of great interest to various industries like the pharmaceutical or the food ones. In this frame, the husks of roasted cocoa beans, that are a by-product of the cocoa processing industry, can constitute a source of pectin. The recovery process has been already defined at laboratory scale with boiling acid extraction (pH 2.5). This process is suitable to recover a quantity of pectin, expressed as anhydro-galacturonic acid (AGA), around 8 g AGA/100 g dry husks; this pectin is characterized by low degree of methylation (%DM around 31) and acetylation degree lower than 2%. In this paper the effects of some operative conditions on pectin quantity and quality were studied, in order to optimize the parameters that can make the process economically competitive: the in-excess quantities of solvents and operation time were reduced, without altering yield and pectin characteristics. In particular, the extract was concentrated by 13.3%, the ethanol for pectin precipitation was reduced (ratio extract to ethanol equal to 1:4), and it was also demonstrated that a single washing with 40% ethanol is sufficient to obtain a purified product.

## 1. Introduction

In 2017 about 100 thousand tons of cocoa beans was imported in Italy, against a world production of 4.6 million tons and about the whole amount is ground [1]. Roasted cocoa beans are still covered by lignocellulosic husks which can be easily removed after roasting itself and before grinding. Considering the large amount of processed beans, the resulting volume of wasted husks is significant and can represent a disposal problem in those districts where there is a high concentration of companies working in the cocoa processing sector.

Reduction, recovery and recycling of organic residues are well known as three of the main environmental challenges for the food industry [2]. In this frame, food processing by-products can be source of valuable molecules, of great interest for the pharmaceutical, cosmetic, and food industries [3, 4].

By this way, different uses of roasted cocoa husks can be found: looking at their chemical and physical characteristics, they are usually sent to combustion or exploited as fiber supplement for animal feeding or used to produce activated carbon [5, 6]. As alternative, husks can be submitted to matter recovery operations becoming a source of additives or other high value products. In particular, at laboratory scale they have been already investigated as a potential source of pectin [4, 5, 7].

Extraction conditions have a relevant influence on the chemical characteristics of the extracted pectin [8–10]. At industrial level, the extraction process is usually done by boiling acid, with hydrochloric acid as the most used (nitric, citric, and oxalic acid are common alternatives). After extraction, pectin precipitation is carried out with high concentration or absolute ethanol or isopropanol. At last, the precipitate is washed with high concentration (> 70%) ethanol in single or multiple operations.

Previous laboratory runs to extract pectin from roasted cocoa husks gave yields between 6.0 and 8.4 g AGA/100 g dry husks, with the maximum yield obtained with boiling HCl solution (pH 2.5) for one hour [7].

Since husks contain a low amount of pectin if compared with traditional sources (e.g., apple pomace and citrus peels), the whole recovery process must be competitive in order to make it attractive for the pectin commercialization. The process defined at laboratory scale utilizes quantities of solvents in excess and long process time. In view of an industrial process, these conditions are not sustainable since they would require high operating costs. For this reason, all the “excess” must be reduced, in terms of quantity of used chemicals and times optimization without changing the yield of extraction.

The operative conditions of the extraction step were widely studied, and scientific literature is rich of optimization data [7–13]. However, less attention was paid to the pectin precipitation and washing conditions, notwithstanding they are time and chemical consuming.

In this frame, the main aim of the present study was to optimize the process “economy”; in particular, the operations to recover and purify the extracted pectin, namely, alcohol precipitation and washing (APW), were investigated and their optimization to save time and chemicals amount was tried, even if they are the most commonly used techniques [8–10, 12].

## 2. Materials and Methods

### 2.1. Raw Materials and Chemicals

For this study husks of Venezuelan roasted cocoa beans were utilized; husks were ground in a grinding mill before pectin extraction.

All the chemicals and reagents were of analytical grade (Sigma Aldrich S.r.l. Milano, Italy). The kit for the enzymatic analysis of acetic acid (Cat. No. 10 148 261 035) was purchased from Boehringer Mannheim/R-BIOPHARM AG, Darmstadt, Germany.

### 2.2. Pectin Extraction and Recovery

A previous study by Mollea et al. [7] defined the procedure for pectin extraction and recovery by means of these steps:

1. extraction with HCl solution at pH 2.5 for 1 hour with ratio husks mass to HCl solution one equal to 1:25;
2. precipitation with 7 volumes of boiling 99.8% ethanol for 20 minutes;
3. one washing with 10 volumes of 63% ethanol.

The modifications studied in this paper are related to the operations no. 2 and no. 3, and they were done in the order indicated in the following subchapters.

### 2.3. Precipitation: Ethanol Quantity Reduction and Contact Time Prolongation

At industrial level, the volume ratio of extract to alcohol may vary from 1:1 to 1:5 even if the ratio 1:3 allows a complete recovery [14].

The first analyzed change was the reduction of the ethanol quantity. As reference, the method by Mollea et al. [7] was considered: this method uses the ratio 1:7. Trials were performed using 4, 2 and 1 volumes of boiling 99.8% ethanol with respect to the extract volume.

The precipitation time was studied, too: the referential time (20 minutes) was increased threefold (1 hour) and sixfold (2 hours) for each extract-to-ethanol ratio. For the ratio 1:1 just the trial with the longest time (2 hours) was carried out. In all the runs, the temperature was 85°C.

### 2.4. Precipitation: Extract Concentration

To minimize the volume of alcohol, the common industrial process usually concentrates the extract before precipitation. Thus, the extract was vacuum concentrated in order to remove partially the solvent by heating at 60°C and constant pressure. The extract was concentrated by 11.5%, 13.3%, and 15.2% with respect to the initial volume; higher concentrations can cause pectin degradation [14].

For each concentrated extract the precipitation was done with ratio extract to 99.8% ethanol equal to 1:4 and precipitation time equal to 20 minutes.

### 2.5. Washing: Number of Operations

In order to control the purity of the final product, the runs with the three aforesaid concentrated extracts were also repeated modifying the washing operation. The pectin was washed twice with 63% ethanol.

The effect of the double washing was evaluated both in quantitative terms (as g AGA/100 g dry husks) to control that the higher washing volume did not cause pectin loss and in qualitative terms (as %DM and acetylation degree) to check impacts on pectin quality.

### 2.6. Washing: Reduction of Ethanol Concentration

To reduce volumes of ethanol, the alcohol concentration in the washing solution was reduced. In subsequent experiments the effect of a lower ethanol concentration was studied in samples with extract concentrated by 13.3% of the starting volume (item 2.4): a single washing was used with the ethanol concentration reduced at 45, 40, and 35%, respectively, instead of 63%. Also in this case quantity and quality of pectin were controlled.

The set of experiments is summarized in Table 1.

### 2.7. Precipitation and Washing: Ethanol Reduction with 13.3% Concentrated Extract

Pectin recovery was carried out with the 13.3% concentrated extract and reducing the ratio extract to ethanol at 1:2 and 1:1 (20 minutes and 1 and 2 hours as precipitation times). The washing operation was single and with 40% ethanol.

### 2.8. Extraction Yield and Pectin Quality

Each experimental run was checked in terms of

- extraction yield, as anhydro-galacturonic acid content (g AGA/100 g dry husks) [15];
- pectin quality, as %DM [16] and acetyl ester content [7];
- presence of polyphenols in solubilized pectin with the *α*-naphthol method [17].

All analyses were carried out in triplicate.

## 3. Results and Discussion

### 3.1. Precipitation: Ethanol Quantity Reduction and Contact Time Prolongation

**Figure 1:**
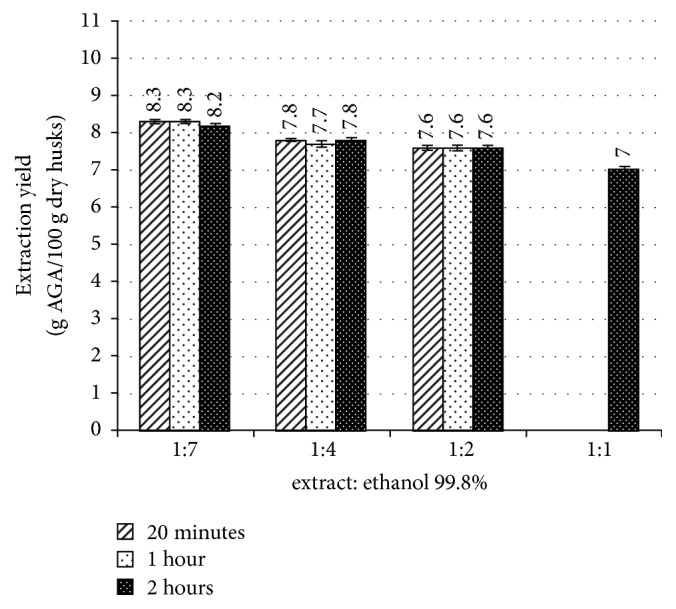
Pectin recovery (as g AGA/100 g dry husks) for the tested extract-to-ethanol ratios. Values are mean and the error bar shows standard deviations for n = 3.

Figure 1 shows the results.

As regards the reduction of ethanol, getting from the ratio 1:7 to 1:4, the quantity of recovered pectin is slightly lower compared to the reference (1:7). When the ethanol quantity is additionally decreased (ratio 1:2), there is another limited decrement which becomes relevant if referred to the 1:7 run. Finally, for the lowest ethanol quantity (1:1), the recovered pectin reduces by more than a percentage point compared to the optimum precipitation conditions.

About the contact time, its prolongation did not give any benefit on the precipitated quantity for a given ratio extract to ethanol (runs with ratios 1:7, 1:4, and 1:2).

On the basis of these results it was decided to choose the ratio 1:4 and precipitation time of 20 minutes: these conditions are a good compromise considering extraction yield and saving of ethanol and processing time, on one side, and pectin loss, on the other. These operative conditions for the precipitation step were kept as reference in the following runs.

As it is shown in Table 2, the simple reduction of the ethanol quantity does not cause alterations of pectin characteristics.

As for pectin obtained with the ratio 1:7, the product recovered with both ratios 1:4 and 1:2 is Low Methoxylated (LM) since its value is around 30. The same conclusion is applicable to the acetylation degree which is always lower than 2%.

### 3.2. Precipitation: Extract Concentration

**Figure 2:**
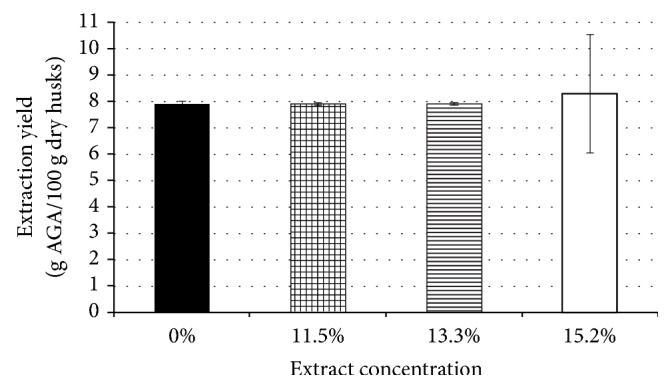
Pectin (as g AGA/100 g dry husks) obtained from extract concentrated at different value (11.5, 13.3, and 15.5 %). Results are mean and the error bar shows standard deviations for n = 3.

Figure 2 shows that recovery yield is not influenced by the extract concentration up to 13.3%. Vice versa, when the extract was concentrated by 15.2%, the average yield resulted apparently higher, but this value is mean from a results set with high variability (over 27% as reported by error bars). The 15.2% concentrate had red color and was not completely transparent due to residual impurities still present after washing and interfering with the procedure utilized for the AGA determination. Other previous studies had already noticed the red color for the extractions with hydrochloric acid at pH 1.5 and 1.0 [7] and with acetic acid at pH 1.6-2 [10, 11]. Therefore, for all the following experiments the 13.3% concentrated extract was utilized, maintaining the ratio extract to ethanol equal to 1:4, while the rest of the procedure was kept unchanged.

### 3.3. Washing: Number of Operations

Considering that the initial extract is impure and impurities can interfere negatively with the final product, it was decided to modify the washing operation by doubling it (all the other operative conditions were not modified).

The results are reported in Table 3, and they show that the number of washings influences neither the quantity of recovered pectin nor the pectin quality. In addition, the test with *α*-naphtol is negative for all the samples, confirming the absence of residual neutral sugars. At the same time, impurities such as polyphenols are absent.

These results demonstrate that two washings are not necessary and that a single operation with 63% ethanol is sufficient to obtain pectin with good purity level.

### 3.4. Washing: Reduction of Ethanol Concentration

For washing, three different concentrations of ethanol were tested, namely, 35, 40, and 45%.

The 35% ethanol caused prolongation of the pectin separation.

Pectin obtained with 40 and 45% ethanol was analyzed in terms of quality and purity, and Table 4 reports the results, as g AGA/100 g dry husks, %DM, and acetylation degree.

The data are very similar to those obtained with 63% ethanol, demonstrating that washing can be operated with 40% ethanol instead of higher concentrations.

## 4. Conclusions

The optimized operative conditions of the process for pectin recovery from roasted cocoa husks can be considered sustainable since good yields were achieved with reduced solvent quantity and working time.

In particular, the study demonstrated that it is possible to reduce ethanol for the precipitation using the ratio extract to ethanol equal to 1:4, while lower ratios give relevant pectin loss.

To reduce the ethanol quantity used for precipitation, the extract can be concentrated by 13.3% under vacuum at 60°C without changing pectin properties.

The precipitation seems to be not affected by time and, for this reason, the minimum duration equal to 20 minutes can be adopted.

At last, considering the purity of the final product, a single washing with 40% ethanol is sufficient.

Except for one condition, the experimental results showed good precision, demonstrated by the standard deviation values. Therefore, the optimized operative conditions can be considered rather reliable.

In the commonly used conditions the ethanol concentration is usually kept high also for washing (over 70%) and with multiple operations (2-3 times). Therefore, comparing them to the ones presented in this paper, the changes showed a good saving of ethanol and processing time.

About its quality, the recovered pectin has low degree of methylation (around 31%) and low degree of acetylation (< 2%), therefore suitable for bakery jams and yogurt (if pectin final uses require higher %DM values, treatments will have to be carried out).

As future perspective, these optimized data can be useful for a preliminary laboratory scale-up.

## Figures and Tables

**Table 1:** Set of experiments done changing: number of washings, ethanol concentration, and extract concentration with respect to the initial volume.

		Extract concentration^*∗*^			
Number of washings	Ethanol concentration	0%	11.5%	13.3%	15.2%
1	63%	X	X	X	X
2	63%	X	X	X	X
1	45%			X	
1	40%			X	
1	35%			X	

^*∗*^With respect to the initial volume.

**Table 2:** Characteristics of pectin obtained with different ratios extract to 99.8% ethanol.

Ratio extract to 99.8% ethanol					
1:7		1:4		1:2	
%DM	Acetylation degree	%DM	Acetylation degree	%DM	Acetylation degree
30.6 ± 0.8	1.6 ± 0.1	31.0 ± 0.5	1.5 ± 0.1	30.8 ± 0.7	1.9 ± 0.1

Values are mean ± standard deviations for n = 3.

**Table 3:** Influence of number of washings on pectin characteristics and extraction yield.

	Extract concentration^*∗*^				
	0%	11.5%	11.5%	13.3%	13.3%
Number of washings	1	1	2	1	2
Extraction yield (g AGA/100 g dry husks)	8.2 ± 0.1	7.9 ± 0.1	8.0 ± 0.1	7.8 ± 0.2	8.1 ± 0.1
%DM	31.7 ± 0.5	31.5 ± 0.6	30.9 ± 0.6	31.0 ± 0.5	30.7 ± 0.8
Acetylation degree	1.4 ± 0.1	1.6 ± 0.1	1.5 ± 0.1	1.9 ± 0.1	1.6 ± 0.1

^*∗*^With respect to the initial volume.

Values are mean ± standard deviations for n = 3.

**Table 4:** Characterization of pectin obtained with different ethanol concentrations in the washing operation.

	Ethanol concentration		
	40%	45%	63%
Extraction yield (g AGA/100 g dry husks)	8.1 ± 0.1	8.0 ± 0.1	7.8 ± 0.2
%DM	32.0 ± 0.6	31.5 ± 0.6	31.0 ± 0.5
Acetylation degree	1.8 ± 0.1	1.9 ± 0.1	1.9 ± 0.1

Values are mean ± standard deviations for n = 3.

## Data Availability

The data used to support the findings of this study are available from the corresponding author upon request.
